# Generating high-fidelity synthetic time-to-event datasets to improve data transparency and accessibility

**DOI:** 10.1186/s12874-022-01654-1

**Published:** 2022-06-23

**Authors:** Aiden Smith, Paul C. Lambert, Mark J. Rutherford

**Affiliations:** 1 Department of Health Sciences, Centre for Medicine, University of Leicester, University Road, Leicester, LE1 7RH UK; 2 Department of Medical Epidemiology and Biostatistics, Karolinska Institutet, Stockholm, Sweden

**Keywords:** Simulation, Survival, Data accessibility, Flexible parametric survival models, Reproducible research, Time-to-event, Synthetic data

## Abstract

**Background:**

A lack of available data and statistical code being published alongside journal articles provides a significant barrier to open scientific discourse, and reproducibility of research. Information governance restrictions inhibit the active dissemination of individual level data to accompany published manuscripts. Realistic, high-fidelity time-to-event synthetic data can aid in the acceleration of methodological developments in survival analysis and beyond by enabling researchers to access and test published methods using data similar to that which they were developed on.

**Methods:**

We present methods to accurately emulate the covariate patterns and survival times found in real-world datasets using synthetic data techniques, without compromising patient privacy. We model the joint covariate distribution of the original data using covariate specific sequential conditional regression models, then fit a complex flexible parametric survival model from which to generate survival times conditional on individual covariate patterns. We recreate the administrative censoring mechanism using the last observed follow-up date information from the initial dataset. Metrics for evaluating the accuracy of the synthetic data, and the non-identifiability of individuals from the original dataset, are presented.

**Results:**

We successfully create a synthetic version of an example colon cancer dataset consisting of 9064 patients which aims to show good similarity to both covariate distributions and survival times from the original data, without containing any exact information from the original data, therefore allowing them to be published openly alongside research.

**Conclusions:**

We evaluate the effectiveness of the methods for constructing synthetic data, as well as providing evidence that there is minimal risk that a given patient from the original data could be identified from their individual unique patient information. Synthetic datasets using this methodology could be made available alongside published research without breaching data privacy protocols, and allow for data and code to be made available alongside methodological or applied manuscripts to greatly improve the transparency and accessibility of medical research.

**Supplementary information:**

The online version contains supplementary material available at 10.1186/s12874-022-01654-1.

## Background

It is common to simulate data with known properties to assess the performance of current and newly developed statistical methods. This approach of simulating data is more complex when the data are a time-to-event nature; with careful thought required on appropriate distributional assumptions and the impact of censoring or competing risks. Often a series of simplistic assumptions are made for the data generating mechanisms, but there have been suggested improvements to create more realistic and complex data, whilst still retaining a known functional form. In the survival analysis framework, this can pertain to simulating biologically plausible data with flexible distributions for both the event censoring times and survival times [1, 2]. Many simulation studies generate data informed by a real-world dataset in order to create realistic scenarios. A further simulation approach is to generate data that closely resembles a given real-world dataset- synthetic data generation. It is this latter approach which is the focus of this manuscript. We aim to generate high-fidelity synthetic data which resembles the covariate patterns and survival times of an existing real-world dataset.

Commonly, journals request authors to release code and data alongside their manuscripts, however this is not always feasible due to data access and privacy constraints [3], with studies in cancer research releasing data in only 11–25% of publications [4–6]. Through synthetic data generation, we can construct datasets which appear and behave in a very similar way as the original data, which can be publicly released as they contain no real-world patient information. Limited access to statistical code is potentially a barrier to open science, methods development and reproducible research [7, 8], and having more openly available data should aid in encouraging this practice.

The synthetic data generation process is split into two key components. We first construct a dataset which replicates the covariate distributions and relationships found in the original data using sequential regression modelling, then use complex survival modelling conditioned on those covariate values to predict synthetic survival times.

In this manuscript, we provide a step by step overview of the methods developed to generate a synthetic dataset which accurately emulates the data structure and survival patterns found in a real-world dataset, as well as providing evidence for choices made regarding the data generation process such as survival model complexity and covariate model ordering. The effectiveness of these methods are appraised by comparing agreement of both all-cause and relative survival metrics estimated in both the original and the synthetic datasets. We implement a distance measure for individual patient date and survival time information to demonstrate the negligible probability that a patient could be re-identified from the synthetic dataset. We discuss the nuances of data privacy, and potential applications to preserve it, in detail with the view to alleviate concerns pertaining to re-identification of individuals from the original data. We also discuss further applications of these methods, as well as potential extensions to make it more broadly utilizable by researchers wishing to disseminate their work with accompanying data and code.

## Methods

### Motivating dataset

The initial data requires a set of core covariates that are prognostic for our disease of interest which will be included in the survival model. In this example focused on cancer survival, we include age, stage and year of diagnosis, a time-to-event variable and an indicator of vital status at the end of follow-up. These covariates are included due to their known influence on survival [9–11]. There is scope to include further patient level information such as deprivation, treatment information or tumor grade and anatomical subsite where applicable [12, 13], depending on data availability and the aims of the analysis. These papers [9–13], like the majority of papers, did not publish data alongside the manuscript due to data confidentiality issues with individual patient data. A potential concern would also be to create data which is so closely correlated, with patients with unique covariate patterns that synthetic individuals would have patient-level information identical to real data individuals. However, survival models have typically low levels of explained variation (R^2^ values) and therefore the chances of fitting a model with such high degrees of correlation is very low [14]. Similarly, in time-to-event data we generally consider low-dimensional data with common covariate groups, and as such the chances of overfitting a model to contain enough parameters to perfectly predict individuals from the real data is improbable. To demonstrate the synthetic data generating methods here, we use a Colon cancer dataset [15] with diagnoses from 1985–1994 of 9,064 patients available from: https://pclambert.net/data/colon.dta (and as a.csv file in the supplementary materials), to create a synthetic dataset from which to estimate a range of all-cause and relative survival metrics. We use freely-available historical data that has previously been used for demonstrative purposes. The data were originally collected in accordance with a legal obligation to report cancer cases to the national population-based register, and the data have been used in the past for illustrative purposes to exemplify statistical methods for the analysis of population-based cancer data.

It is necessary to recover the distributions of all covariates included in the survival model, here: stage at diagnosis, sex, age-at-diagnosis and anatomical subsite. To reconstruct diagnosis date, which derives the exit date and the administrative censoring date, we must accurately replicate the distribution of year at diagnosis within the whole dataset. The synthetic survival times are predicted from a centile distribution of survival times conditional on the generated synthetic individuals’ covariate pattern.

All data generation methods and statistical analyses here were implemented using Stata 17 [16] with code provided as supplementary material, but could also be applied in other statistical software such as R using equivalent modelling commands.

Table 1 outlines the 12 steps required to execute the simulation process for generating synthetic data, each of which will be expanded on subsequently.

**Table 1:** Overview of simulation process to construct replicated time-to-event datasets

Simulation Methods:
1.Clean and format the original data which is going to be replicated
2.Define administrative censoring date and create an individual exit variable before assigning data as time-to-event
3.Assign new factor levels for variables with missing data groups, generate dummy variables, and generate any required non-linear and interaction terms for the survival model
4.Fit sequentially increasing complex models for individual covariates and store model estimates from which to recover simulated covariate distributions
5.Fit an all-cause survival model, including between-covariate interactions and time-dependent effects
6.Set a seed and number of observations for the replica data, and using the stored model estimates for each covariate model, sequentially generate covariate values in the replica data based on conditional values of earlier covariates in the sequence
7.Recreate any non-linear effects and model interactions which were included in the original survival model
8.Use post estimation predict option from the survival model to generate synthetic survival times based on individual patient covariate patterns from the stored survival model estimates
9.Format vital status variable and generate diagnosis date and exit date variables
10.Re-format vital status variable using exit date and the administrative censoring date to reconstruct the original data censoring distribution
11.Clean and label all simulated variables
12.Assign the simulated data as time-to-event for use in future survival analysis

### Data cleaning and formatting (Step 1, Table 1)

We require certain covariates in our initial data in order to generate synthetic data of sufficient fidelity. In this example our initial dataset contains: calendar year of diagnosis (1985–1994), stage at diagnosis (“Localized”, “Regional”, “Distant” and “Missing”), sex (Male or Female), anatomical subsite (“Coecum and Ascending”, “Transverse”, “Sigmoid and Descending” and “Other and NOS”), age at diagnosis (18–99), vital status at time of censoring (Dead or Alive), unique patient id number, cancer diagnosis date and exit date (diagnosis date + survival time in days, administratively censored). We include covariates such as anatomical subsite, which is not necessarily included in the final analysis model, but incorporates additional heterogeneity to increase survival variation above and beyond that which is derived solely from the covariates which are included in the analysis model [17]. The initial distributions of these covariates are shown in the first column of Table 2*.*

**Table 2:** Comparison of original data and simulated data covariate distributions

	**Original Data (%)**	**Simulated Data (%)**	**Absolute Difference (%)**
*Stage at Diagnosis*			
Localized	3716 (40.91)	3724 (41.28)	0.37
Regional	1148 (12.64)	1140 (12.64)	0.00
Distant	2907 (32.00)	2836 (31.46)	0.54
Missing	1313 (14.45)	1319 (14.62)	0.17
*Anatomical Tumour Subsite*			
Coecum and Ascending	3239 (35.66)	3227 (35.77)	0.11
Transverse	1607 (17.69)	1569 (17.39)	0.30
Sigmoid and Descending	3660 (40.29)	3659 (40.56)	0.27
Other and NOS	578 (6.36)	566 (6.27)	0.07
*Age Group*			
< 45	379 (4.17)	368 (4.08)	0.09
45–60	1338 (14.73)	1448 (16.05)	1.32
60–75	3699 (40.72)	3604 (39.95)	0.77
> 75	3688 (40.38)	3601 (39.91)	0.47
*Sex*			
Male	3799 (41.82)	3724 (41.28)	0.54
Female	5285 (58.18)	5297 (58.72)	0.54
*Vital Status*			
Alive	3557 (39.16)	3467 (38.43)	0.73
Dead	5527 (60.84)	5554 (61.57)	0.73

### Defining date information (Step 2, Table 1)

Following data cleaning, step 2 states we must define an administrative censoring date from the original data to be used in the final simulated dataset. This is defined as the last observed date of follow-up found in the original data, here this is 31/12/1995. Using this censoring date, we create an exit date variable to help define our data as time-to-event, constructed by adding the survival time in days to the diagnosis date for every individual in the dataset. If an individual’s last date of follow-up exceeds the censoring date, is it reassigned to be equal to our administrative censoring date.

### Data preparation for the survival model (Step 3, Table 1)

Step 3 of the simulation process requires the construction of dummy variables for our factor variables which will be included in the survival model (stage, subsite, sex), following the reassignment of missing data as its own factor level. Here, we have missing data in stage at diagnosis. To replicate patterns of missingness, we assign missing values as an additional level in the covariates of interest and treat them as their own unique group. The necessity of this process depends on the intended use for the data. If the data is being used to assess imputation methods, it is paramount to have missing data simulated as accurately as possible, if the data is solely for testing analysis methods it becomes less important.

### Model fitting for covariate pattern recovery (Step 4, Table 1)

To draw survival time predictions from the fitted survival model, it is necessary to first accurately recapture the joint distribution of the covariates which inform the survival model. Step 4 involves creating separate models for each covariate to model the joint distribution using a series of conditional distributions which are sequentially more complex, with example code provided below. For the process of simulating covariate values for individual $i$ for the $M$ variables of interest, we fit $M$ regression models, with each variable, $m\left(=1..M\right),$ being the outcome for one of the $M$ models. The form of the ${m}^{th}$ regression model is dependent on the variable type for $m$ (categorical, continuous, etc.). We start with a base model with no other covariates to get the marginal distribution of our first covariate of interest (age in the example below). We then include any prior variables $(1,\dots ,m-1)$ in the process in all subsequent models; effectively with the final ${M}^{th}$ model containing all the other variables and corresponding interaction terms. We include both the main covariate effects and the interaction effects between the specified covariates. Factor variables are fitted using a multinomial logistic regression model, and fitted in order of least-to-most distributional complexity such that the most complex covariates are modelled with the most information. For continuous covariates we propose a linear regression model with spline terms for an inverse-normal-rank-based-transformation of the covariate of interest in order to approximate continuous covariates with non-normal distributions. The ranks of the original covariate are converted to a normal distribution [18] and then we flexibly model the relationship between the original covariate and the inverse rank-normal values using restricted cubic splines to allow for a non-normally shaped distribution to be captured. Increased flexibility could be incorporated with an increased number of degrees of freedom for the rank splines, however this can be sensitive and requires user specification.

Continuous covariates could also be simulated by specifying skewness and kurtosis values to allow non-normal distributions or a transformation applied to approximate normality. The effectiveness of each method will vary depending on the data, but we have found the method of using the restricted cubic splines based on the ranks to be robust to a range of distributions for the original covariate (see Supplementary Material 2).

### Fitting the survival model (Step 5, Table 1)

The 5^th^ step is to fit a survival model. The survival time predictions we later generate are derived from the parameters from a flexible parametric survival model [19], implemented using stpm2 in Stata [20]. These models are highly flexible and allow for non-proportional hazards. The use of restricted cubic spline functions allows for far more complex hazard functions than in standard parametric models. We model age (and more generally all continuous covariates) using restricted cubic splines to allow for non-linear effects, and use Winsorizing for age, below the 2^nd^ percentile and above the 98^th^ percentile of the age distribution to aid with model fitting [21]. Two-way interaction terms are generated and included in the survival model for the age splines and stage at diagnosis, anatomical subsite and sex. Age and stage at diagnosis are all included as time-dependent effects. The overall model is fitted with 5 degrees of freedom, and 3 degrees of freedom for each time-dependent effect. We model these survival times flexibly to appropriately account for the fact that covariate effects are likely to wane in the long-term. Higher order interactions could be implemented, but care must be taken when increasing the complexity of models fitted as privacy becomes more of a concern as complexity, and by extension fidelity, increases.

An important consideration is the level of modelling complexity required. We can generate from a simple survival model or a very complex one, dependent on what we aim to gain from the data. A simple model with no time-dependent effects and no interactions will give broadly similar marginal all-cause survival estimates, however when estimating more complex metrics, or looking at comparisons within missing data groups, the complex model may provide estimates more similar to that which was present in the original data.

When considering model selection we require some level of prior knowledge of the dataset, and this relates to the complexity of the analysis model that the synthetic data is required for. For example, if we were to release code with a paper that is considering a complex survival model, then this model complexity must be inherent and reflected within the data generation process. If we only require a simple model, we can generate data from less complex models which also alleviates some data privacy concerns. This flexibility makes these methods generalizable to reflect the purpose of the data, however it does take some degree of fine tuning and decisions on models to fit and therefore being familiar with the data is beneficial.

### Recovering covariate distributions and survival model terms (Step 6 & 7, Table 1)

**Fig. 1:**
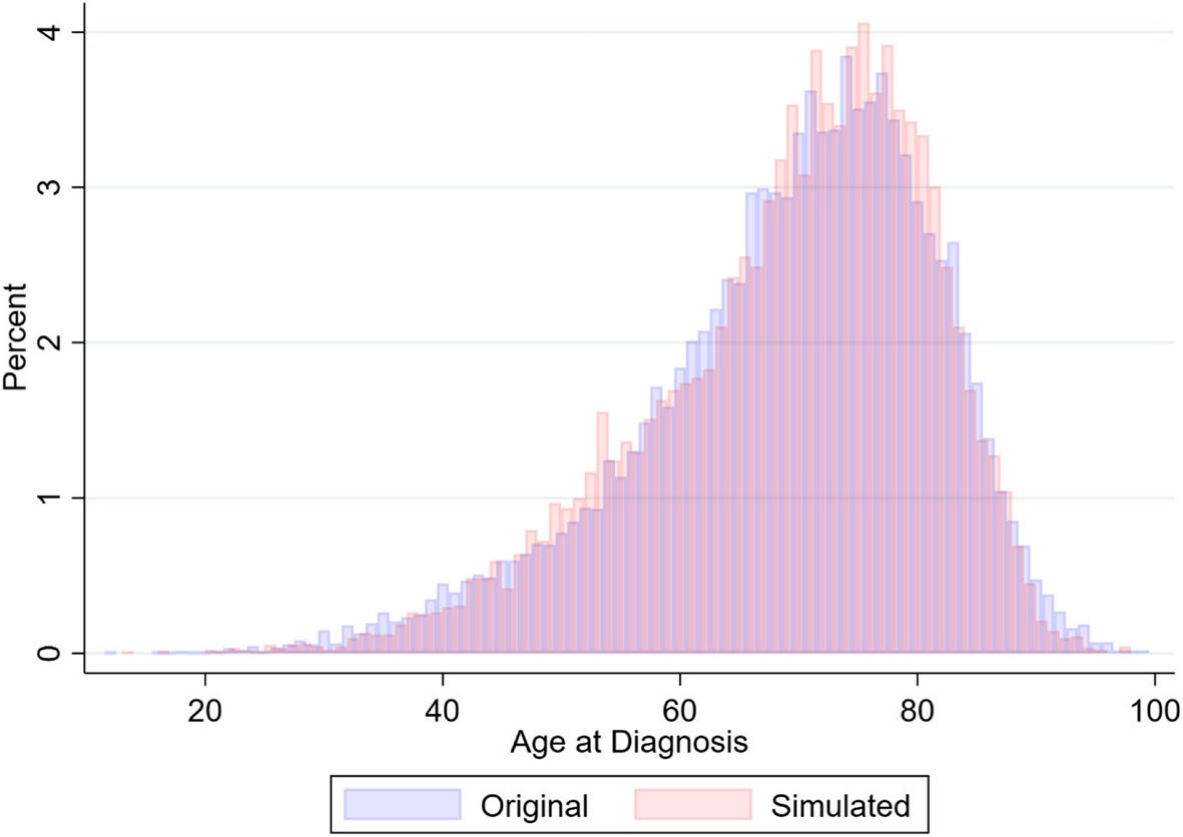
Comparison of original data age-at-diagnosis distribution with synthetic data age-at-diagnosis distribution derived using the Inverse-Normal-Rank-Based transformation method

Having modelled our covariate distributions and fitted the survival model from which to draw predictions, we now start with a blank dataset from which to recreate the data based on the stored parameter estimates from models fitted to the original data. Step 6 requires setting the simulation seed and number of observations to be generated, the covariate distributions can be recreated by sampling from the stored covariate models. The covariate models are restored in the same order the models were fitted to ensure all model predictions have the necessary information. For factor variables direct probabilities define the proportions in each group. Figure 1 demonstrates the effectiveness of using the inverse-normal-rank-based transformation with the age distribution being recovered accurately due to the increased flexibility away from a rigid normal distribution (see Supplementary Material 2 for further demonstration of the effectiveness of this method across various distributions).

The age splines, and any interaction terms fitted in the original survival model also need to be recreated prior to drawing survival predictions from the stored model estimates, per step 7.

### Generating simulated survival times (Step 8, Table 1)

Step 8 generates survival times from the overall survival model fitted to the original data, using the inversion methods where a random uniform (0,1) variable is transformed to a survival time using Brent root finding methods. This is implemented using the stpm2 post-estimation predict command, where individual survival times conditional on patient covariate patterns are generated. Because each individuals’ covariate pattern is synthesized from a distributional range rather than a specific real-world individual, we do not model survival times directly from a true individual, and therefore there is no link between a synthetic individual and any individual from the real-world dataset.

### Formatting vital status and date information (Step 9, Table 1)

Step 9 codes vital status using the last observed exit time from the original data as a threshold for if an event (death) has been experienced for any given individual within the datasets follow-up period. Survival times are rounded up to the nearest full day.

To reconstruct diagnosis date, month and day of diagnosis are generated randomly and uniformly across the calendar year, reflecting the patterns of diagnosis found in the original data. For disease areas where diagnoses are seasonal, month of diagnosis could be treated the same as year of diagnosis by more precisely recreating the distribution of diagnoses across the year. However, cancer is not a disease in which diagnoses varies seasonally, so in this example this process was not necessary.

### Replicating administrative censoring in the simulated data (Step 10, Table 1)

Step 10 calculates survival time in days in order to define an exit date for all synthetic patients, and then administrative censoring is implemented using a final date of follow-up taken from the original data (31/12/1995). Further vital status coding occurs using the generated exit times, with patients labelled as alive if their exit dates exceed the final date of follow-up. Here we use a combination of calendar year and the administrative censoring date to replicate the censoring distribution found in the original data. For this method of synthetic data generation this is sufficient to accurately capture the known distribution (*see* Table 2), rather than modelling censoring directly through a Weibull or exponential distribution [22]. We generate a model based time-to-event distribution, and artificially construct our time-to-censoring distribution, whereas other simulation methods require model based estimates of both distributions.

### Cleaning and preparing simulated data for survival analysis (Step 11 & 12, Table 1)

Step 11 involves basic data cleaning, such as replacing assigned missing observations as truly missing data for stage at diagnosis, labelling covariates to match the original data and removing any unnecessary variables which have been created during the data generation process. Finally, in step 12 we declare our data as time-to-event in preparation for survival analysis.

### Data privacy

The nature of synthetic data, especially in high-fidelity scenarios, attracts important questions regarding continued security of individual patient data. Broadly, it is difficult to fully conceptualize all aspects of what data privacy truly means, and how to ensure it. In this scenario, we consider the notion of data privacy to pertain to continued confidentiality and security of individuals’ health data [23]. In this context, we require high-fidelity data to promote meaningful information sharing with appropriate utility levels to reproduce research and test methods, while making data available alongside published work to encourage open scientific discourse. In time-to-event scenarios, patient prognosis and outcomes are highly reflective of their covariate profile, and as such to develop any meaningful methodology it is necessary to preserve all of the relationships found within the real-world data.

An unavoidable aspect of synthetic data generation is the inherent trade-off between privacy and utility [24]. We can construct low-fidelity data which reflects the structure of the source data but preserves none of the relationships and can contain implausible covariate values. This data poses no risk of information disclosure, but has little utility beyond understanding how the data is formatted. High-fidelity synthetic data by nature poses a higher risk of information disclosure, however using these methods the risk is still minimal. In time-to-event data, often datasets will have few explanatory variables, non-unique covariate patterns and unexplained heterogeneity within those covariate patterns and therefore re-identification is unlikely to be a genuine risk. In a scenario where every individual had a truly unique covariate profile, we could perfectly predict each individual rendering this methodology inappropriate, however in practice this would not occur. Here, synthetic individuals are generated from a series of regression models each with its own inherent unexplained variation, then further generating random date variables within calendar years which further ensures no synthetic individuals are genuine reflections of any individual from the real dataset.

To demonstrate the preservation of individual patient data privacy using these methods, we consider a range of patients from the original data with rare or unique covariate patterns and compare their individual-level date and survival time information to 1 million synthetic patients directly generated to have a matched covariate profile.

### Appraisal metrics

Following the construction of the synthetic data the recovery of the covariate distributions will be evaluated against the original dataset. Comparisons of all-cause survival across the length of follow-up, and 5-year relative survival will be made between the true data and the synthetic data. In population-based cancer studies, it is typical to report relative survival estimates. Relative survival estimates are used to try to isolate the mortality that is directly associated with the diagnosis of cancer by removing the impact of deaths due to causes other than cancer. This is achieved by incorporating general population background mortality rates, making it possible to isolate the theoretical survival that is only attributable to the disease being investigated [25, 26]. The accuracy of the synthetic data here is evaluated across a range of metrics.

The consistency of a marginal 10-year relative survival estimates is compared with the true data across 25 different simulated datasets, and an age-specific life expectancy measure is also compared between the true data and 10 simulated datasets.

## Results

### Covariate distribution recovery

It is necessary to replicate covariate distributions found in the original data with a high degree of accuracy in order to ensure that survival predictions being made conditional on unique covariate patterns are reflected appropriately in the synthetic data.

For factor covariates simulated from multinomial logistic regression models (stage at diagnosis, anatomical tumour subsite, sex, year of diagnosis), the absolute difference in factor level proportions between the original and simulated datasets never exceeds 1% (see Table 2). Similarly, there is only discrepancy of 0.84 percentage points in the proportion of subjects recorded as being dead.

In this example, the simulated data has a higher proportion of 45–60 year olds by 1.32 percentage points. As demonstrated in Fig. 1, this redistribution of individuals does not vastly affect the overall age distribution being reconstructed.

### Demonstrating survival pattern replication

The effectiveness of these methods in replicating survival patterns found in the original data can be demonstrated by comparing various survival estimates for the original and synthetic data. Marginal all-cause survival estimates stratified by population subgroups are compared, as well as a model based estimate of 5-year relative survival.

**Fig. 2:**
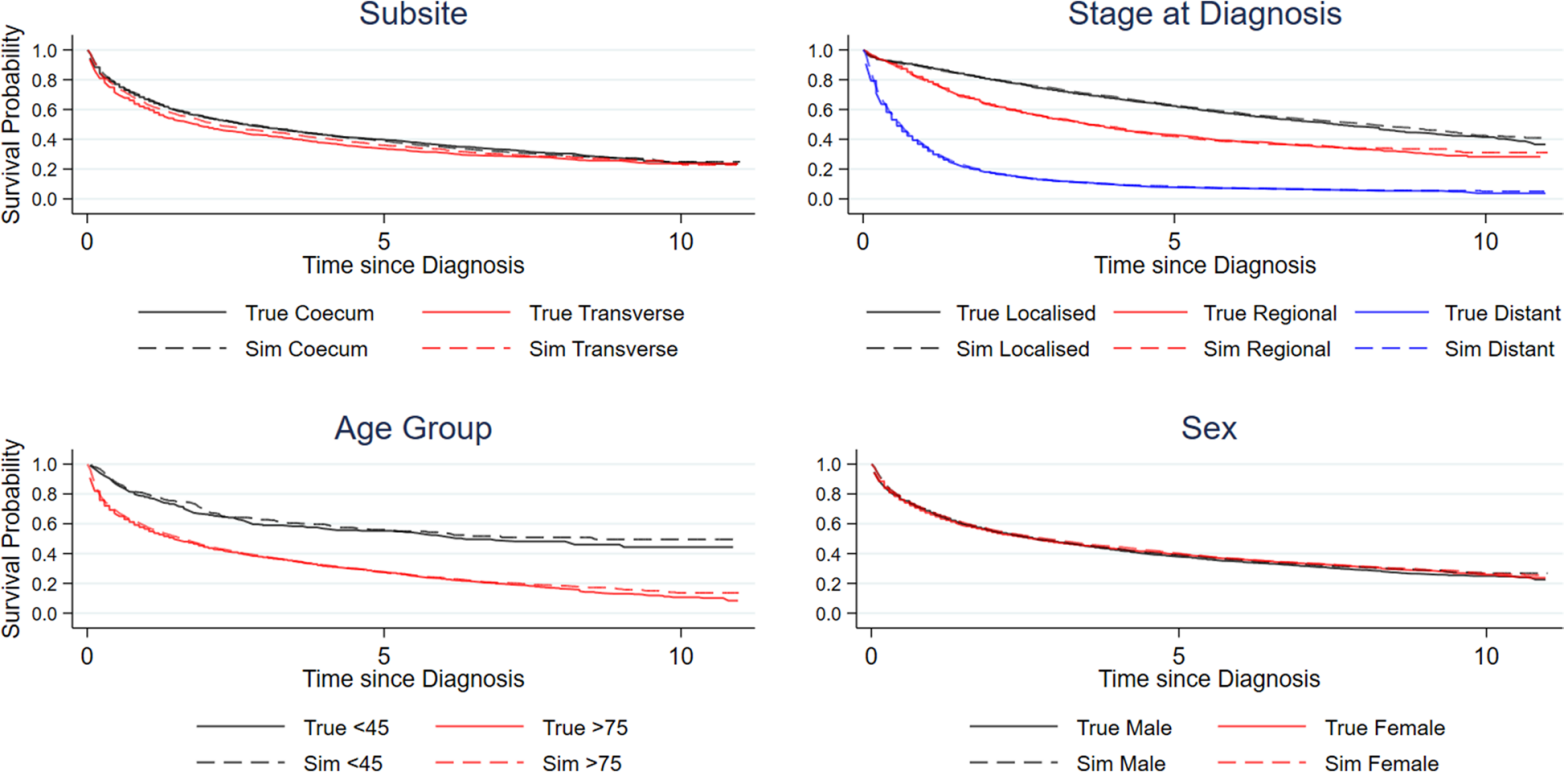
Marginal all-cause survival estimates stratified by population subgroups (Subsite: Transverse and Coecum; Age Group: Under 45 and Over 75; Stage: Localized, Regional and Distant; Sex: Male and Female) comparing true estimates from the original and replicated estimates in the synthetic data

Figure 2 demonstrates the replication of the marginal all-cause survival estimates stratified by anatomical subsite (Coecum and Transverse), age group (under 45 s and over 75 s), stage at diagnosis and sex. There is some slight deviation in agreement in the age group subgroups, however it is still close enough to be broadly comparable when working with the synthetic data.

**Fig. 3:**
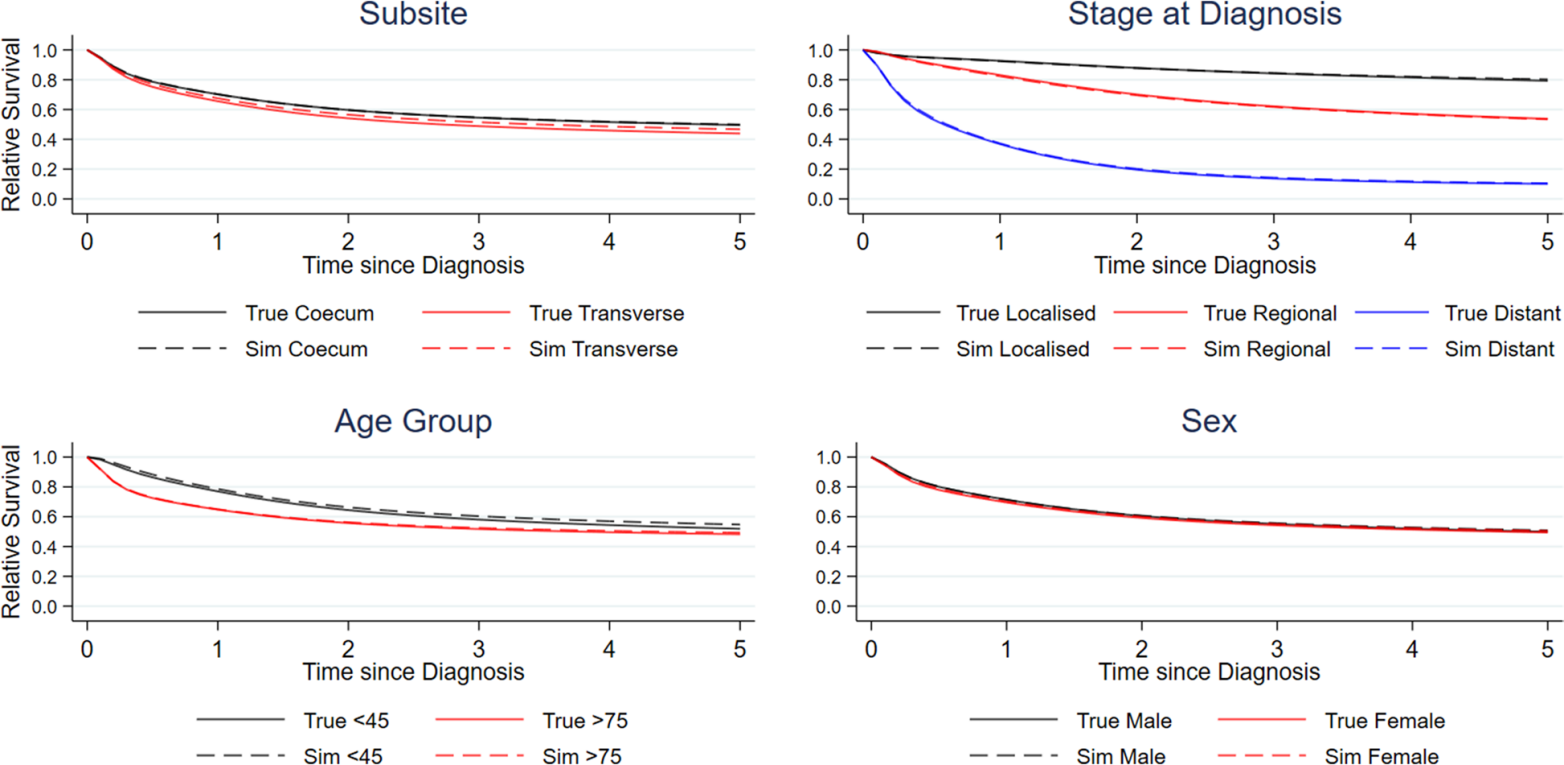
5-year relative survival estimates stratified by population subgroups (Subsite: Transverse and Coecum; Age Group: Under 45 and Over 75; Stage: Localized, Regional and Distant; Sex: Male and Female) comparing original and synthetic data

Figure 3 demonstrates the agreement in relative survival estimates over time since diagnosis stratified across various patient level variables. Overall, there is good agreement between the synthetic data and the original data, only marginally overestimating survival in some groups. This demonstrates that the methods generate good agreement for both all-cause and relative survival, despite being generated from an all-cause survival model.

**Fig. 4:**
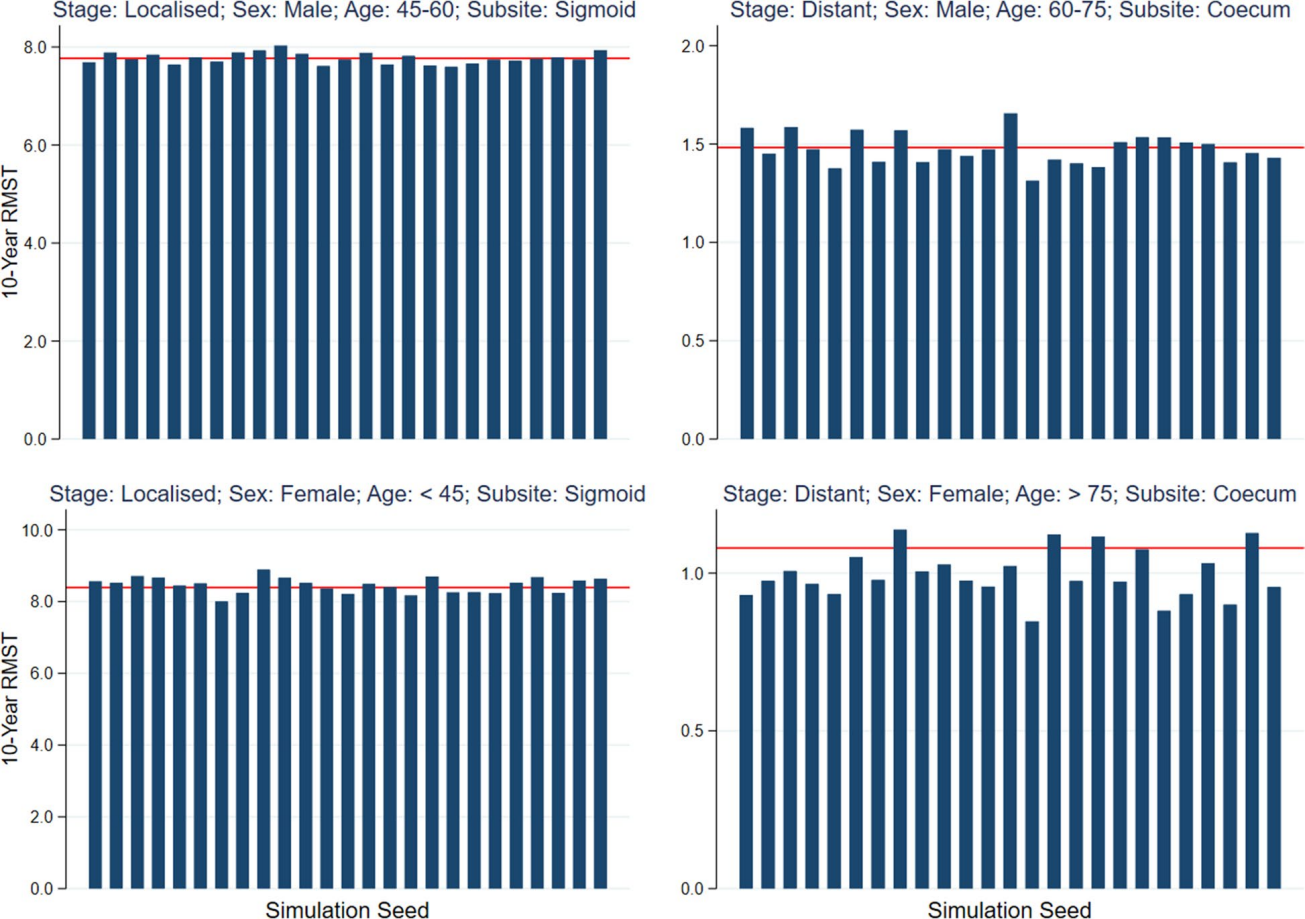
Marginal 10-year relative survival predictions estimated across assorted covariate patterns (Top Left: Stage: Localized, Sex: Male, Age Group: 45–60, Subsite: Sigmoid. Top Right: Stage: Distant, Sex: Male, Age Group: 60–75, Subsite: Coecum. Bottom Left Stage: Localized, Sex: Female, Age Group: < 45, Subsite: Sigmoid. Bottom Right: Stage: Distant, Sex: Female, Age Group: > 75, Subsite: Coecum.) for a range of 25 simulation seeds to demonstrate synthetic data consistency. (True prediction from original data shown as red horizontal line)

Marginal 10-year restricted mean survival time is estimated for varying covariate patterns across 25 different simulation seeds and the distribution of the metric examined to compare point estimates. Individual conditional life expectancy estimates are also compared across simulation seeds for males across a range of ages and stages at diagnosis. Figure. 4 demonstrates the consistency of the synthetic data at closely replicating survival estimates regardless of the simulation seed specified in the code. Across 25 different seeds, 10-year restricted mean survival time predictions have been estimated and compared with the corresponding prediction made in the original dataset.

**Fig. 5:**
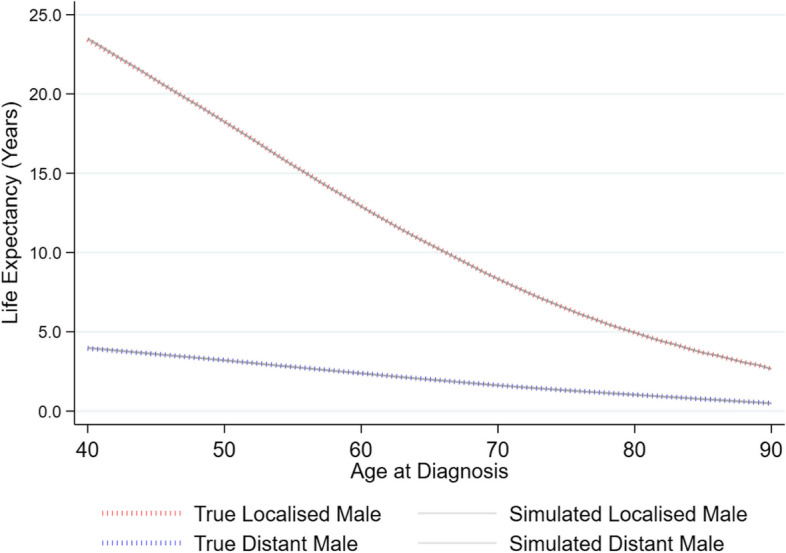
Conditional Life expectancy estimated across a range of ages (40–90) for males with either a localized or distant cancer stage at diagnosis comparing original and synthetic data (10 different simulation seeds)

Figure 5 demonstrates the effectiveness of the synthetic data at replicating a conditional life expectancy metric where extrapolation of survival times beyond the known follow-up in the data is necessary. This metric is predicted for individual males diagnosed with both localized and distant stage cancer across ages ranging from 40–90, with the survival patterns replicated accurately when compared to the same metric in the original data.

### Model specifications

Two key questions for constructing the synthetic dataset pertained to the complexity of survival model required to accurately represent individual survival times conditional on patient covariate patterns, and the ordering of covariates in the sequential conditional models used to recreate the overall joint distribution of covariates within the original dataset. The synthetic datasets here have been expanded to 1,000,000 observations in order to eliminate differences in survival patterns due to random variation.

**Fig. 6:**
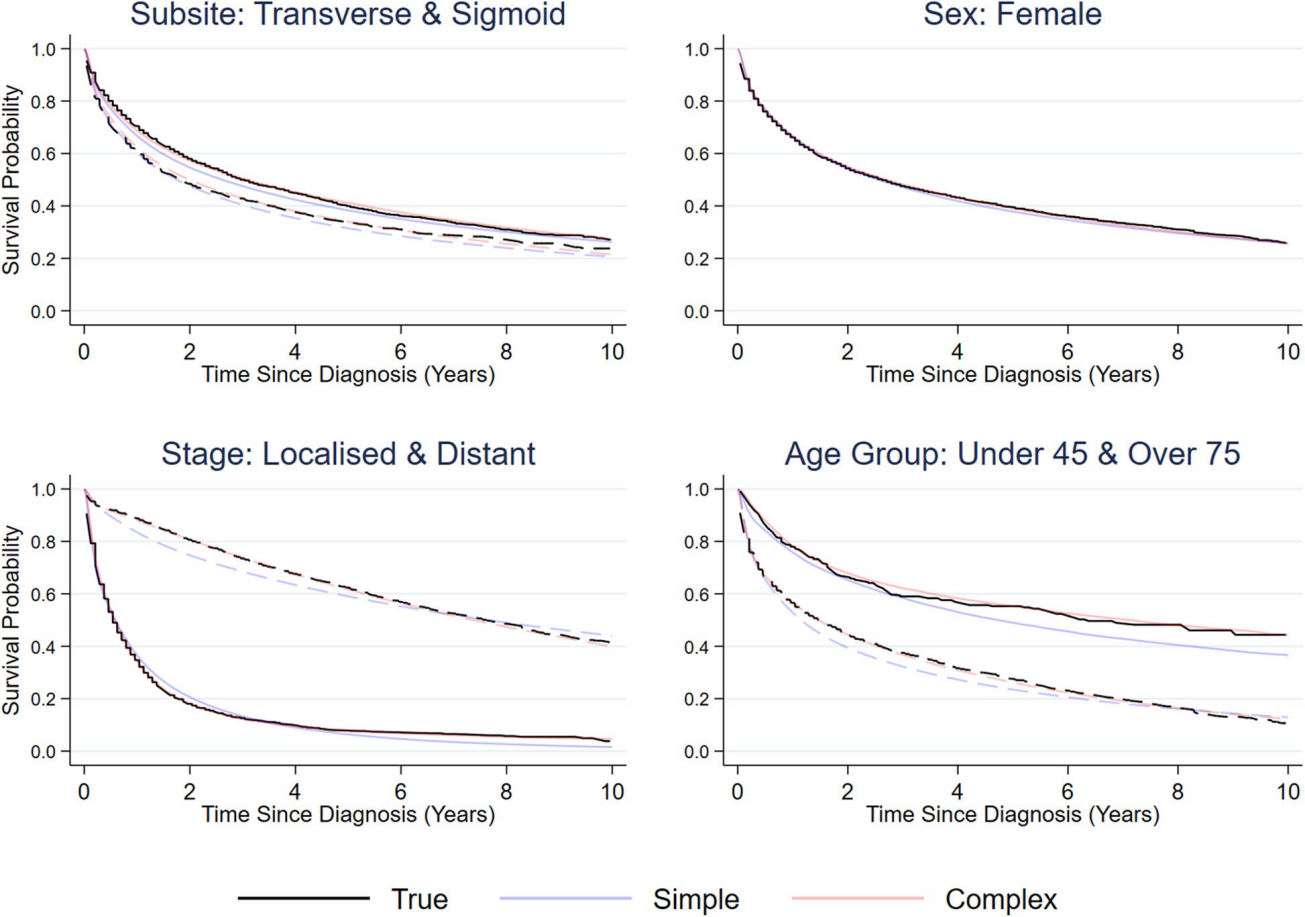
Kaplan–Meier estimates with marginal predictions in the corresponding population subgroups (Subsite: Transverse (dashed lines) and Sigmoid; Age Group: Under 45 and Over 75 (dashed lines); Stage: Localized (dashed lines) and Distant; Sex: Female), from two flexible parametric models of differing complexity compared with equivalent estimates from the original data

**Fig. 7:**
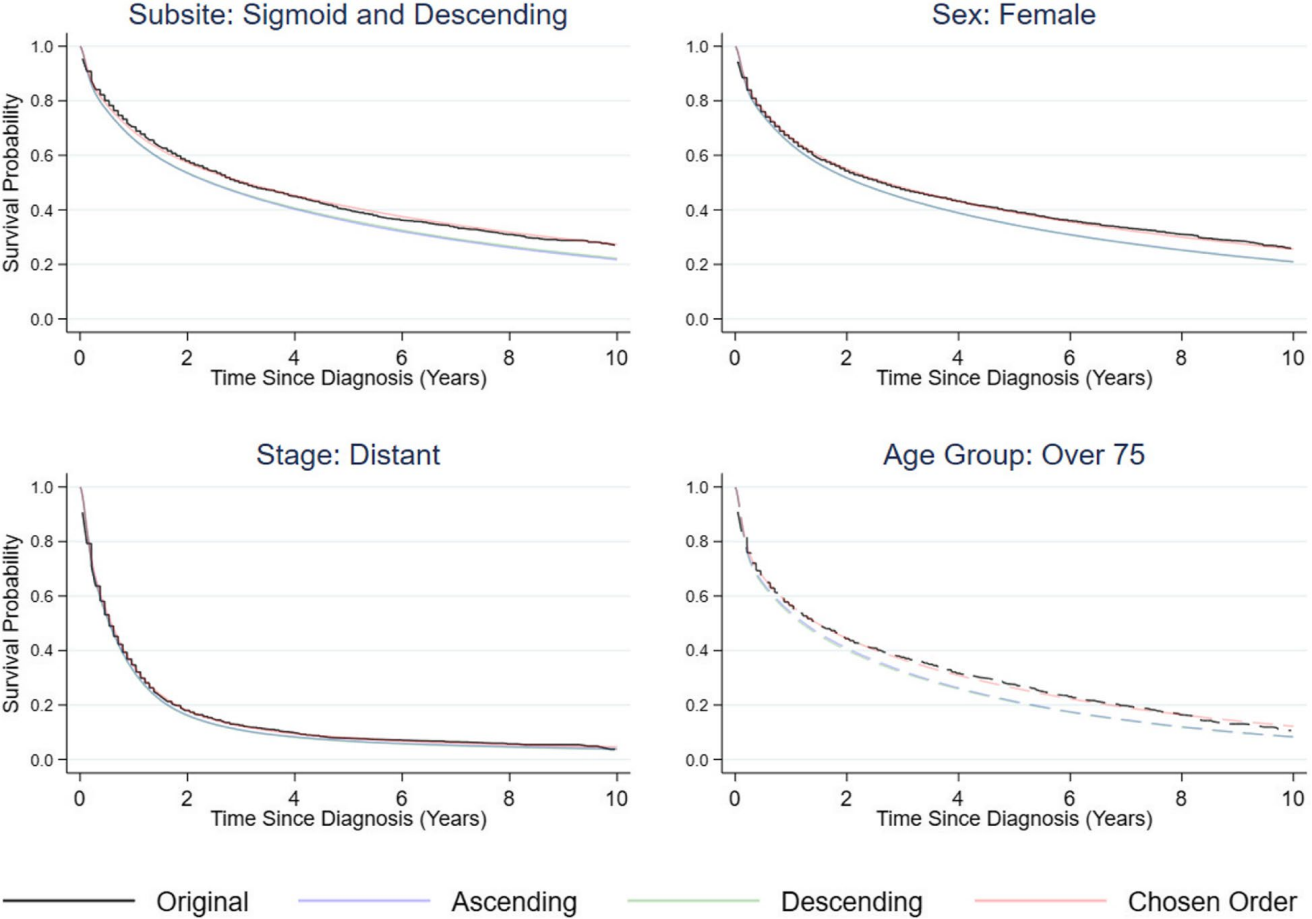
Kaplan–Meier estimates with marginal predictions in the corresponding population subgroups (Subsite: Sigmoid; Age Group: Over 75; Stage: Distant; Sex: Female), where covariate distributions are modelled in different orders. Ascending Order: Sex, Stage, Subsite, Year of Diagnosis; Descending Order: Year of Diagnosis, Subsite, Stage, Sex; Chosen Order: Year of Diagnosis, Stage, Sex, Subsite)

Figure 6 shows that across all population subgroups it is beneficial to include added complexity, through interaction terms, to best capture survival patterns across all unique covariate patterns. The simple model includes only main effects for stage, subsite, sex and age, without consideration of interactions and time-varying effects. The complex model builds on the simple model by including interactions between stage and age, stage and sex, subsite and age, and finally sex and age. It also includes time-varying effects for age and stage. In this example, the covariate order which is most consistent with the true survival estimates is as follows: Age, Year of Diagnosis, Stage at Diagnosis, Sex, and Anatomical Subsite. This order, as demonstrated in Fig. 7, provides more accurate all-cause survival estimate regeneration compared to ordering the covariates from most to least categorical levels and vice versa. The ordering of covariate modelling gives the best predictive accuracy when covariates are modelled from least to most distributional complexity. By modelling the most complex distribution last, we feed the model with the most information in in order to capture the shape of that distribution most effectively. Modelling the continuous covariates first allows for the effect of a continuous variable to be imparted on each factor variable model, aiding in preservation of interrelationships. The similarity of these covariate distributions in comparison to the original data is shown in Table 2*.* More detailed cross-tabulations of covariate distribution recovery can be seen in Supplementary Material  1.

### Non-replication of exact patient information

A significant concern with generating patient level information to replicate survival patterns from a real-world dataset is the issue of patient confidentiality. Despite there being no link between a single synthetic and real-world individual, it may be necessary to convince data controllers that the synthetic data is still not identifiable. Utilization of synthetic data is still in its infancy, and as such there is still skepticism and lack of understanding which inhibits the wider acceptance of these synthetic datasets in practice. Using this synthetic data generation method, there are very small probabilities that any single uniquely measured covariate pattern and corresponding survival time and date information will be replicated for any single individual within the dataset. Here, we identify a series of real-world individuals with very rare or unique covariate patterns in our dataset (*see* Table 3) and create a synthetic dataset for each of them consisting of 1 million synthetic individuals.

**Table 3:** Comparison of survival time and diagnosis date matching when comparing a rare real-world individual to 1 million synthetic individuals with matched covariate profiles

	***Real-World Individual Covariate Patterns***					
*No of Observations*	1	3	3	2	1	1
*Age at Diagnosis*	68	73	81	70	60	45
*Sex*	Female	Male	Female	Female	Male	Male
*Stage at Diagnosis*	Localised	Localised	Distant	Regional	Distant	Localised
*Year of Diagnosis*	1994	1994	1992	1990	1993	1989
*Anatomical Subsite*	Other	Sigmoid	Coecum	Coecum	Transverse	Sigmoid
*Vital Status*	Alive	Alive Alive Alive	Dead Dead Dead	Dead Alive	Dead	Alive
Synthetic Vital Status (Alive/Dead%)	94.74/5.25	89.36/10.64	9.92/90.08	44.07/55.93	17.22/82.78	78.44/21.56
*Diagnosis Date*	15/9/1994	14/1/1994 15/10/1994 15/9/1994	21/12/1992 17/3/1992 15/4/1992	20/11/1990 16/5/1990	7/12/1993	14/11/1989
*Synthetic Observations with Diagnosis Date* ± *15 Days (%)*	8.65	7.75 8.32 8.57	7.01 8.36 8.68	8.56 8.37	8.42	8.56
*Survival Time (Rounded to Nearest Day)*	472	716 442 472	16 46 868	351 2055	77	2238
*Synthetic Observations with Survival Time* ± *15 Days (%)*	8.43	6.74 4.10 8.10	11.61 15.77 0.45	1.61 3.77	7.26	7.01
*Survival Time Matches per 1 Million Synthetic Patients*	2719	2282 2573 2633	4102 2187 156	2740 2670	1011	2225

We evaluate the diagnosis date and survival time of individuals with rare or unique covariate patterns against 1 million synthetic observations generated which are forced to have an identical covariate profile. We also assess the vital status distributions of the synthetic individuals generated.

Due to the random generation of month and day of diagnosis to construct our synthetic diagnosis date, we expect that a proportion of synthetic diagnosis dates will be within a given range due to chance. Given in this scenario the year of diagnosis is fixed, we would expect approximately 8.3% (or 83,000) of the 1 million synthetic individuals to have a diagnosis date within a 30-day range of the real-world individual. This point is demonstrated in Table 3. Further to this, when evaluating the vital status distributions, there is still discrepancies from the real individual, even where a single individual is considered. This demonstrates there is no direct link to the real-world individual and the synthetic information is generated as a result of a distributional probability.

We also generated 1000 synthetic datasets of equal size to the original data, and counted the frequency at which the synthetic covariate patterns investigated in Table 3 occur. In no instance do these synthetic covariate patterns, which are rare in the original data, occur in all of the synthetic datasets generated. For truly unique covariate patterns, the covariate pattern occurred in 42% or less of the 1000 datasets. This is before any date information or survival time is considered, and so these individuals will be even further anonymized from any original individual. This further indicates that we are generating synthetic individuals from probability distributions rather than directly taking individuals from the original data and generating replicas.

The variation in survival time predicted from the model is subject to wider variation in predicted values because of the lack of explained variation in the survival model, even in the given scenario where an individual has a unique covariate profile. We see matching survival times (when rounded to the nearest day), however this is only due to the chance of the true value being in the plausible value range of the centile distribution for the specific covariate pattern. If times were not rounded, we would see greatly reduced numbers, if any, of exact matching survival times. This further highlights that on the individual level, our synthetic data does not aim to accurately recreate a patient from the original dataset, but instead focuses on generating data with plausible distributions across population subgroups to capture the patterns found in the real data.

## Discussion

The synthetic data generation methods presented in this paper describe a way to accurately generate time-to-event data through the use of flexible parametric survival models. By generating covariate distributions which reflect those found in the original data, we can estimate survival times predicted from distributions specific to any synthetic individuals covariate profile. Time-to-event data is not routinely considered in synthetic data generation, where financial and consumer data is a more prevalent research area. Health data, and by extension time-to-event data, is uniquely nuanced and so care must be taken to capture this data in such a way that preserves the utility of the dataset. Time-to-event data in a registry style setting is unique in that we must consider the combination of general censoring and the application of administrative censoring dates. We therefore need to generate high-fidelity synthetic data across calendar time, and apply a date of entry and exit which is plausible based not only on the covariate profile of the individual, but also in terms of the general incidence patterns and exit date from the original data.

Current synthetic data methods focus mainly on artificial intelligence and machine learning methods [27–30], which despite continued growth, are still not widely understood by all researchers. We feel that the simplicity of these methods where a basic understanding of regression modelling and survival models is all that is required, as well as methods to directly address the specific issues of time-to-event data, provides a simple and easily implementable method of generating synthetic data with sufficient fidelity to maintain high data utility without compromising patient data privacy and provides a unique contribution to the literature.

Alternative methods to de-identify data include suppression, generalization, date offsetting, and noise injection [31]. Generating a dataset using the methods presented here does not pose a significant risk to re-identification of individuals from the original data, while still maintaining the overall structure and internal distributions.

Previous literature regarding simulation of survival data tends to focus on the creation of entirely new data under a range of set parameters [1, 2, 31–33]. Many simulation studies make use of either exponential or Weibull distributions [34, 35], however these are often not flexible enough to fully capture the shape of underlying hazard functions found in real-world clinicals trial or population based data, where at least one turning point is observed in the hazard function [21]. Making use of the flexibility in a Royston-Parmar model provides a good solution for replicating real-world survival data. We are aware of one other statistical package for directly generating synthetic versions of data, *synthpop* [36], however this uses a combination of parametric and classification and regression tree (CART) methods to accurately recreate covariate distributions, and is only currently available in R.

Significant time was dedicated to conceptualising and putting into practice notions of data privacy and how best to preserve them. We considered other common data privacy methods such as differential privacy [37] and k-anonymity [38]. K-anonymity does not include any true observational modifications to the data, and therefore potential data attackers can still make logical inferences from the data which may compromise individuals. Similarly, this method can potentially skew the results of a dataset if data is supressed or generalised disproportionately, therefore decreasing the utility of the data and rendering it unsuitable for the purposes previously described. Differential privacy assumes a “worst-case scenario” where the data attacker already has information from the original data. In practice, with healthcare data this is an unlikely scenario given the rigorous protective measures already in place for the real data. Even so, because there is no relationship between a synthetic and real-world individual, combined with mostly low-sparsity covariate patterns, it is highly unlikely the synthetic data poses a risk of re-identification. Given the majority of health data is already anonymised, we believe that the synthetic data methods provide a second layer of de-identification from a real-world individual by predicting from distributions rather than attempting to directly generate exact individuals from the original data.

Similarly, we considered generating values from categorisations of continous covariate prior to randomly assigning a continous value within that category as an extra privacy layer, however based on our results we feel in this instance it is unecessary, but provides a potential solution in other contexts. It would also be possible to reduce the complexity of the covariate and survival models being fitted, for example by not incorporating interaction effects, as a way to generate synthetic data if the reason for the data generation is purely for illustrative purposes alongside a manuscript. This is likely to remove more complex relationships found in the original data, and could be used as another layer of data privacy if full data utility wasn’t required in a particular circumstance. Using these methods, we feel it is unecessary to implement further data privacy methods, and the risk of identifying an individual from the real-world data within the synthetic data is minimal.

We consider these methods to be easily adaptable to most time-to-event datasets, given only a set of covariates and an initial understanding of which covariates are prognositic for the disease of interest is necessary. The provided code in the supplementary file provides the direct methodology to recreate the synthetic data from this paper, and can be easily altered to generate synthetic data from a different real-world data source. A further consideration for the application of these methods is within smaller datasets such as clinical trials. Here we are demonstrating the efficacy of these methods in a dataset of approximately 9000 patients, and specific adaptations and considerations for smaller datasets will form part of future work.

A development of the example data we use here would be the inclusion of an increased number of covariates. To include additional covariates it is necessary to not only recover their distributions, but also include them in the survival model if we are interested in survival differences within that covariate. In doing so, we increase the complexity of the model significantly with each additional model parameter. It is important to balance increasing model complexity without overfitting the model [39]. In real-world cancer registry data there is scope for much more detailed patient information, treatment information and molecular information, all of which could be incorporated using this methodology should it be required by the user. Any method which is effective at predicting survival could be used, for example data with high covariate to observation structure could benefit from lasso type modelling with shrinkage. Future work will extend into a competing risks framework.

Despite comparing relative survival estimates on the generated data, we originally fitted an all-cause model in the data generation procedure. We did so because of complications found when predicting from the relative survival model in certain settings, particularly when the relative survival curves for a specific covariate pattern reached a plateau. This led to unstable predicted values of the survival times because of the influence of the plateau on the survival time predictions, which are based on centiles of the covariate-specific survival distribution.

We handled factor variables with missing data by assigning the missing group as a separate individual level of the variable so that the patterns of missingness could be preserved and recovered in the simulation process. Throughout this paper we have shown that there is a negligible possibility for an individual from the original data to be identified within the synthetic data, we have shown the all-cause and relative survival estimates are consistently accurate when compared to equivalent estimates made in the original data, and the covariate distributions are recovered accurately throughout.

## Conclusions

The approach that we have outlined allows the possibility for realistic synthetic individual patient data to be made available while simultaneously protecting patient confidentiality. We have focused on the construction of data that are typical in population-based cancer survival research, but the ideas are transferrable to other time-to-event settings. Our proposal to closely capture the distribution of continuous, but non-normal covariates has great flexibility. The benefits of making data available with corresponding code to run the main analyses of a published paper are numerous; allowing reproducible research, offering greater transparency and enabling extensions by other researchers.

Some research suggests that synthetic data can be used as a proxy for the real dataset in analyses [35, 40]. We believe that the synthetic data constructed here is accurate enough to make predictions consistent with the real data, but do not advise that it currently be used in place of the real data as a proxy. We wish to address barriers with synthetic data by encouraging clear lay term explanations of synthetic data, increasing awareness and understanding of synthetic data and its potential, and alleviate potential concerns stakeholders may have with implementing synthetic data generation on real-world data.

## Supplementary information

**Additional file 1. **Supplementary Material 1: Original vs Simulated Covariate Distributions

**Additional file 2.**

## Data Availability

The datasets generated and analyzed during this study are included in this published article and its supplementary information files. The data used here is freely-available for download at the following address: https://pclambert.net/data/colon.dta
