# Supplementary material for: Generating high-fidelity synthetic time-to-event datasets to improve data transparency and accessibility
Source: BMC Med Res Methodol. 2022 Jun 23;22:176. doi: 10.1186/s12874-022-01654-1 (PMC9229142; doi:10.1186/s12874-022-01654-1)
Supplement: Supplementary file 1 — Additional file 1. Supplementary Material 1: Original vs Simulated Covariate Distributions [file 12874_2022_1654_MOESM1_ESM.docx]

**Supplementary Material 1: Original vs Simulated Covariate Distributions**

Table 1: Vital Status Distributions Recovery across Patient Sex

|  | Original | | Total  (Row %)  (Column %) | Simulated | | Total  (Row %)  (Column %) |
| --- | --- | --- | --- | --- | --- | --- |
|  | Male  (Row %)  (Column %) | Female  (Row %)  (Column %) |  | Male  (Row %)  (Column %) | Female  (Row %)  (Column %) |  |
| Alive | 1476 (41.50)  (38.85) | 2081 (58.50)  (39.38) | 3557 (100)  (39.16) | 1438  (41.48)  (38.61) | 2029  (58.52)  (38.30) | 3467  (100) (38.43) |
| Dead | 2323 (42.03)  (61.15) | 3204 (57.97)  (60.62) | 5527 (100)  (60.84) | 2286  (41.16)  (61.39) | 3268  (58.84)  (61.70) | 5554  (100)  (61.57) |
| Total | 3799 (41.82)  (100) | 5285  (58.18)  (100) | 9084 (100)  (100) | 3724  (41.28)  (100) | 5297  (58.72)  (100) | 9021  (100)  (100) |

Table 2: Stage at Diagnosis Distribution across Patient Sex

|  | Original | | Total  (Row %)  (Column %) | Simulated | | Total  (Row %)  (Column %) |
| --- | --- | --- | --- | --- | --- | --- |
|  | Male  (Row %)  (Column %) | Female  (Row %)  (Column %) |  | Male  (Row %)  (Column %) | Female  (Row %)  (Column %) |  |
| Localised | 1573  (42.33)  (41.41) | 2143 (57.67)  (40.55) | 3716  (100)  (40.91) | 1606 (43.13)  (50.05) | 2118  (56.87)  (39.98) | 3724  (100)  (41.28) |
| Regional | 480 (41.81)  (12.63) | 668 (58.19)  (12.64) | 1148 (100)  (12.64) | 471 (41.32)  (14.68) | 669  (58.68) (12.63) | 1140  (100)  (12.64) |
| Distant | 1231 (42.35)  (32.40) | 1676 (57.65)  (31.71) | 2907 (100)  (32.00) | 1132 (39.89)  (30.40) | 1706  (60.11)  (32.21) | 2838  (100) (31.46) |
| Missing | 511 (39.22)  (13.56) | 798 (60.78)  (15.10) | 1313 (100)  (14.45) | 515 (39.04)  (13.83) | 804  (60.96)  (15.18) | 1319  (100)  (14.62) |
| Total | 3799 (41.82)  (100) | 5285 (58.18)  (100) | 9084 (100)  (100) | 3724 (41.28)  (100) | 5297  (58.72)  (100) | 9021  (100)  (100) |

Table 3: Stage at Diagnosis Distribution across Vital Status

|  | Original | | Total  (Row %)  (Column %) | Simulated | | Total  (Row %)  (Column %) |
| --- | --- | --- | --- | --- | --- | --- |
|  | Alive  (Row %)  (Column %) | Dead  (Row %)  (Column %) |  | Alive  (Row %)  (Column %) | Dead  (Row %)  (Column %) |  |
| Localised | 2247 (60.47)  (63.17) | 1469  (39.53)  (26.58) | 3716  (100)  (40.91) | 2216  (59.51)  (63.92) | 1508  (40.49)  (27.15) | 3724  (100)  (41.28) |
| Regional | 514 (44.77)  (14.45) | 634 (55.26)  (11.47) | 1148 (100)  (12.64) | 486  (42.63)  (14.02) | 654  (57.37)  (11.78) | 1140  (100)  (12.64) |
| Distant | 285  (9.80)  (8.01) | 2622 (90.20)  (47.44) | 2907 (100)  (32.00) | 270  (9.51)  (7.79) | 2568  (90.49)  (46.24) | 2838  (100)  (31.46) |
| Missing | 511  (38.92)  (14.37) | 802 (61.08)  (14.51) | 1313 (100)  (14.45) | 495  (37.53)  (14.28) | 824  (62.47)  (14.84) | 1319  (100)  (14.62) |
| Total | 3557  (39.16)  (100) | 5527 (60.84)  (100) | 9084 (100)  (100) | 3467  (38.43)  (100) | 5554  (61.57)  (100) | 9021  (100)  (100) |

Table 4: Age Group Distribution across Vital Status

|  | Original | | Total  (Row %)  (Column %) | Simulated | | Total  (Row %)  (Column %) |
| --- | --- | --- | --- | --- | --- | --- |
|  | Alive  (Row %)  (Column %) | Dead  (Row %)  (Column %) |  | Alive  (Row %)  (Column %) | Dead  (Row %)  (Column %) |  |
| < 45 | 204  (53.83)  (5.74) | 175  (46.17)  (3.17) | 379  (100)  (4.17) | 219  (59.51)  (6.32) | 149  (40.49)  (2.68) | 368  (100)  (4.08) |
| 45-60 | 717  (53.59)  (20.16) | 621  (46.41)  (11.24) | 1138  (100)  (14.73) | 747  (51.59)  (21.55) | 701  (48.41)  (12.62) | 1448  (100)  (16.05) |
| 60-75 | 1627  (43.98)  (45.74) | 2072 (56.02)  (37.49) | 3699  (100)  (40.72) | 1570  (43.56)  (45.28) | 2034  (56.44)  (36.62) | 3604  (100)  (39.95) |
| > 75 | 1009  (27.51)  (28.37) | 2659 (72.49)  (48.11) | 3668 (100)  (40.38) | 931  (25.85)  (26.85) | 2670  (74.15)  (48.07) | 3601  (100)  (39.91) |
| Total | 3557  (39.16)  (100) | 5527 (60.84)  (100) | 9084 (100)  (100) | 3467  (38.43)  (100) | 5554  (61.57)  (100) | 9021  (100)  (100) |

Table 5: Anatomical Subsite Distribution across Vital Status

|  | Original | | Total  (Row %)  (Column %) | Simulated | | Total  (Row %)  (Column %) |
| --- | --- | --- | --- | --- | --- | --- |
|  | Alive  (Row %)  (Column %) | Dead  (Row %)  (Column %) |  | Alive  (Row %)  (Column %) | Dead  (Row %)  (Column %) |  |
| Coecum and Ascending | 1282 (39.58)  (36.04) | 1957  (60.42)  (35.41) | 3239  (100)  (35.66) | 1242  (38.49)  (35.82) | 1985  (61.51)  (35.74) | 3227  (100)  (35.77) |
| Transverse | 561 (34.91)  (15.77) | 1046 (65.09)  (18.93) | 1607  (100)  (17.69) | 541  (34.48)  (15.60) | 1028  (65.52)  (18.51) | 1569  (100)  (17.39) |
| Sigmoid and Descending | 1487  (40.63)  (41.80) | 2173 (59.37)  (39.32) | 3660 (100)  (40.29) | 1493  (40.80)  (43.06) | 2166  (59.20)  (39.00) | 3659  (100)  (40.56) |
| Other and NOS | 227 (39.27)  (6.38) | 351 (60.73)  (6.35) | 578  (100)  (6.36) | 191  (33.75)  (5.51) | 375  (66.25)  (6.75) | 566  (100)  (6.27) |
| Total | 3557  (39.16)  (100) | 5527 (60.84)  (100) | 9084 (100)  (100) | 3467  (38.43)  (100) | 5554  (61.57)  (100) | 9023  (100)  (100) |

Table 6: Stage at Diagnosis Distribution across Anatomical Subsite (Simulated Data)

| ***Simulated***  ***Data*** | Anatomical Subsite  (Row %)  (Column %) | | | | Total |
| --- | --- | --- | --- | --- | --- |
|  | Coecum & Ascending | Transverse | Sigmoid & Descending | Other and NOS |  |
| Localised | 1313  (35.26)  (46.79) | 637  (17.11)  (40.60) | 1618  (43.45)  (44.22) | 156  (4.19)  (27.56) | 3724  (100)  (41.28) |
| Regional | 497  (43.60)  (17.71) | 179  (15.70)  (11.41) | 403  (35.35)  (11.01) | 61  (5.35)  (10.78) | 1140  (100)  (12.64) |
| Distant | 996  (35.10)  (35.50) | 555  (19.56)  (35.37) | 1130  (39.82)  (30.88) | 157  (5.53)  (27.74) | 2838  (100)  (31.46) |
| Missing | 421  (31.92)  (13.05) | 198  (15.01)  (12.62) | 508  (38.51)  (13.88) | 192  (14.56)  (33.92) | 1319  (100)  (14.62) |
| Total | 3227  (35.77)  (100) | 1569  (17.39)  (100) | 3659  (40.56)  (100) | 566  (6.27)  (100) | 9021  (100)  (100) |

Table 7: Stage at Diagnosis Distribution across Anatomical Subsite (Original Data)

| ***Original***  ***Data*** | Anatomical Subsite  (Row %)  (Column %) | | | | Total |
| --- | --- | --- | --- | --- | --- |
|  | Coecum & Ascending | Transverse | Sigmoid & Descending | Other and NOS |  |
| Localised | 1323  (35.60)  (40.85) | 612  (16.47)  (38.08) | 1621  (43.62)  (44.29) | 160  (4.31)  (27.68) | 3716  (100)  (40.91) |
| Regional | 505  (43.99)  (15.59) | 190  (16.55)  (11.82) | 399  (34.76)  (10.90) | 54  (4.70)  (9.34) | 1148  (100)  (12.64) |
| Distant | 1005  (34.57)  (31.03) | 597  (20.54)  (37.15) | 1146  (39.42)  (31.31) | 159  (5.47)  (27.51) | 2907  (100)  (32.00) |
| Missing | 406  (30.92)  (12.53) | 208  (15.84)  (12.94) | 494  (37.62)  (13.50) | 205  (15.61)  (35.47) | 1313  (100)  (14.45) |
| Total | 3239  (35.66)  (100) | 1607  (17.69)  (100) | 3660  (40.29)  (100) | 578  (6.36)  (100) | 9084  (100)  (100) |

Table 8: Stage at Diagnosis Distribution across Age Group (Simulated Data)

| ***Simulated***  ***Data*** | Age Group  (Row %)  (Column %) | | | | Total |
| --- | --- | --- | --- | --- | --- |
|  | < 45 | 45-60 | 60-75 | > 75 |  |
| Localised | 160  (4.30)  (43.48) | 575  (15.44)  (39.71) | 1547  (41.54)  (42.92) | 1442  (38.72)  (40.04) | 3724  (100)  (41.28) |
| Regional | 75  (6.58)  (20.38) | 224  (19.65)  (15.47) | 428  (37.54)  (11.88) | 413  (36.23)  (11.47) | 1140  (100)  (12.64) |
| Distant | 109  (3.84)  (29.62) | 490  (17.27)  (33.84) | 1164  (41.01)  (32.30) | 1075  (37.88)  (29.85) | 2838  (100)  (31.46) |
| Missing | 24  (1.82)  (6.52) | 159  (12.05)  (10.98) | 465  (35.25)  (12.90) | 671  (50.87)  (18.63) | 1319  (100)  (14.62) |
| Total | 368  (4.08)  (100) | 1448  (16.05)  (100) | 3604  (39.95)  (100) | 3601  (39.92)  (100) | 9021  (100)  (100) |

Table 9: Stage at Diagnosis Distribution across Age Group (Original Data)

| ***Original***  ***Data*** | Age Group  (Row %)  (Column %) | | | | Total |
| --- | --- | --- | --- | --- | --- |
|  | < 45 | 45-60 | 60-75 | > 75 |  |
| Localised | 141  (3.79)  (37.20) | 553  (14.88)  (41.33) | 1577  (42.44)  (42.63) | 1445  (38.89)  (39.39) | 3716  (100)  (40.91) |
| Regional | 70  (6.10)  (18.47) | 208  (18.12)  (15.55) | 467  (40.68)  (12.63) | 403  (35.10)  (10.99) | 1148  (100)  (12.64) |
| Distant | 128  (4.40)  (33.77) | 431  (14.83)  (32.21) | 1204  (41.42)  (32.55) | 1144  (39.35)  (31.19) | 2907  (100)  (32.00) |
| Missing | 40  (3.05)  (10.55) | 146  (11.12)  (10.91) | 451  (34.35)  (12.19) | 676  (51.49)  (18.43) | 1313  (100)  (14.45) |
| Total | 379  (4.17)  (100) | 1338  (14.73)  (100) | 3699  (40.72)  (100) | 3668  (40.38)  (100) | 9084  (100)  (100) |

Table 10: Anatomical Subsite Distribution across Age Group (Simulated Data)

| ***Simulated***  ***Data*** | Age Group  (Row %)  (Column %) | | | | Total |
| --- | --- | --- | --- | --- | --- |
|  | < 45 | 45-60 | 60-75 | > 75 |  |
| Coecum & Ascending | 120  (3.72)  (32.61) | 498  (15.43)  (34.39) | 1210  (37.50)  (33.57) | 1339  (43.35)  (38.85) | 3227  (100)  (35.77) |
| Transverse | 59  (3.76)  (16.03) | 290  (18.48)  (20.03) | 636  (40.54)  (17.65) | 584  (37.22)  (16.22) | 1569  (100)  (17.39) |
| Sigmoid & Descending | 170  (4.65)  (46.20) | 596  (16.29)  (41.16) | 1529  (41.79)  (42.43) | 1364  (37.28)  (37.88) | 3659  (100)  (40.56) |
| Other and NOS | 19  (3.36)  (5.16) | 64  (11.31)  (4.42) | 229  (40.46)  (6.35) | 254  (44.88)  (7.05) | 566  (100)  (6.27) |
| Total | 368  (4.08)  (100) | 1448  (16.05)  (100) | 3604  (39.95)  (100) | 3601  (39.92)  (100) | 9021  (100)  (100) |

Table 11: Anatomical Subsite Distribution across Age Group (Original Data)

| ***Original***  ***Data*** | Age Group  (Row %)  (Column %) | | | | Total |
| --- | --- | --- | --- | --- | --- |
|  | < 45 | 45-60 | 60-75 | > 75 |  |
| Coecum & Ascending | 133  (4.23)  (36.15) | 434  (13.40)  (32.44) | 1219  (37.64)  (32.95) | 1449  (44.74)  (39.50) | 3239  (100)  (35.66) |
| Transverse | 83  (5.16)  (21.90) | 239  (14.87)  (17.86) | 652  (40.57)  (17.63) | 633  (39.39)  (17.26) | 1607  (100)  (17.69) |
| Sigmoid & Descending | 135  (3.69)  (35.62) | 606  (16.56)  (45.29) | 1603  (43.80)  (43.34) | 1316  (35.96)  (35.88) | 3660  (100)  (40.29) |
| Other and NOS | 24  (4.15)  (6.33) | 59  (10.21)  (4.41) | 225  (38.93)  (6.08) | 270  (46.71)  (7.36) | 578  (100)  (6.36) |
| Total | 379  (4.17)  (100) | 1338  (14.73)  (100) | 3699  (40.72)  (100) | 3668  (40.38)  (100) | 9084  (100)  (100) |
