# Supplementary material for: Generating high-fidelity synthetic time-to-event datasets to improve data transparency and accessibility
Source: BMC Med Res Methodol. 2022 Jun 23;22:176. doi: 10.1186/s12874-022-01654-1 (PMC9229142; doi:10.1186/s12874-022-01654-1)
Supplement: Supplementary file 2 — Additional file 2. [file 12874_2022_1654_MOESM2_ESM.docx]

**Supplementary Material 2: Simulating Age Distributions**

Figure 1: Demonstration of age distribution reconstruction methods for assorted distributions (Normal, Bimodal. Skewed and Uniform) where age is not dependent on other variables


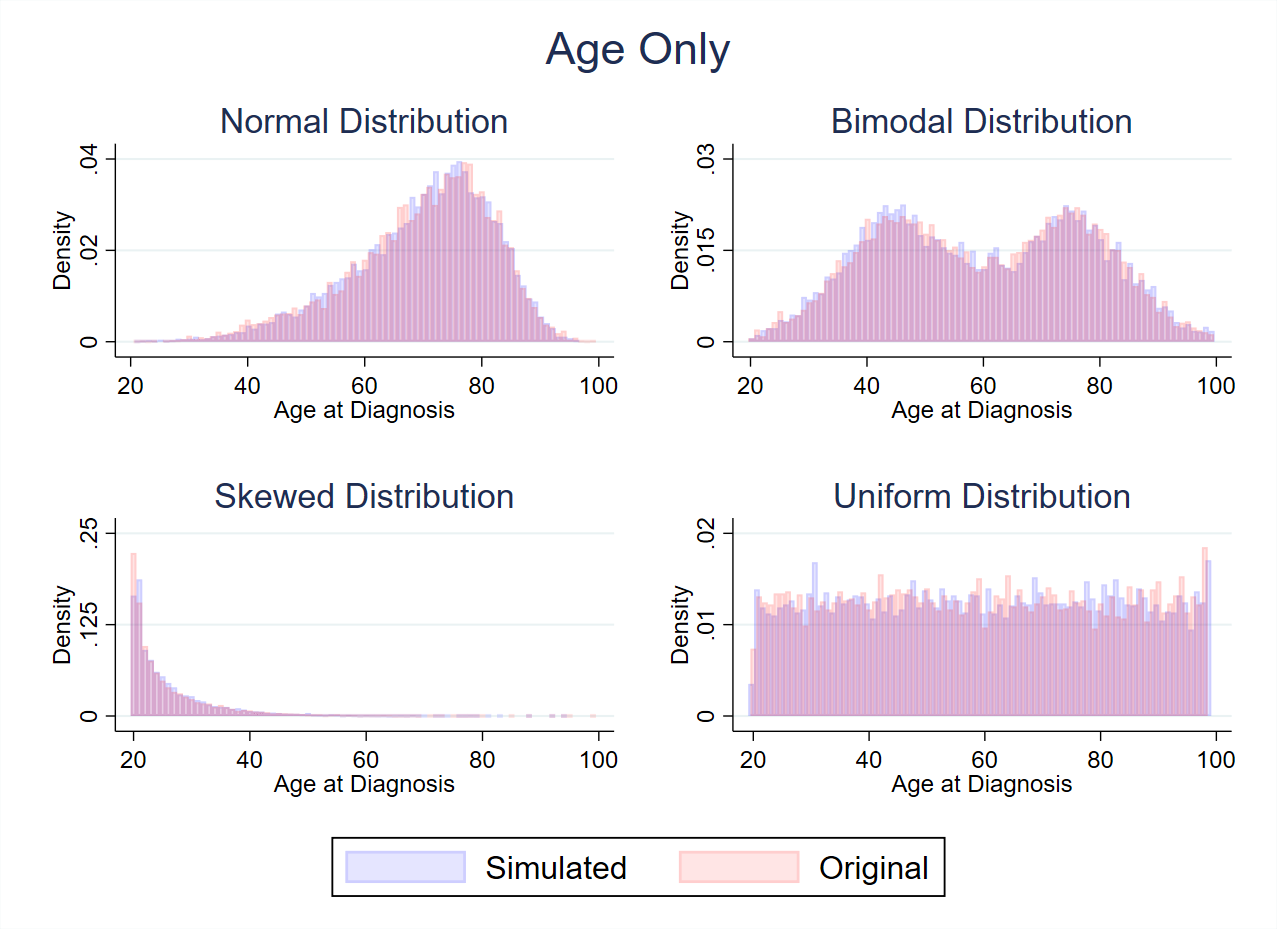

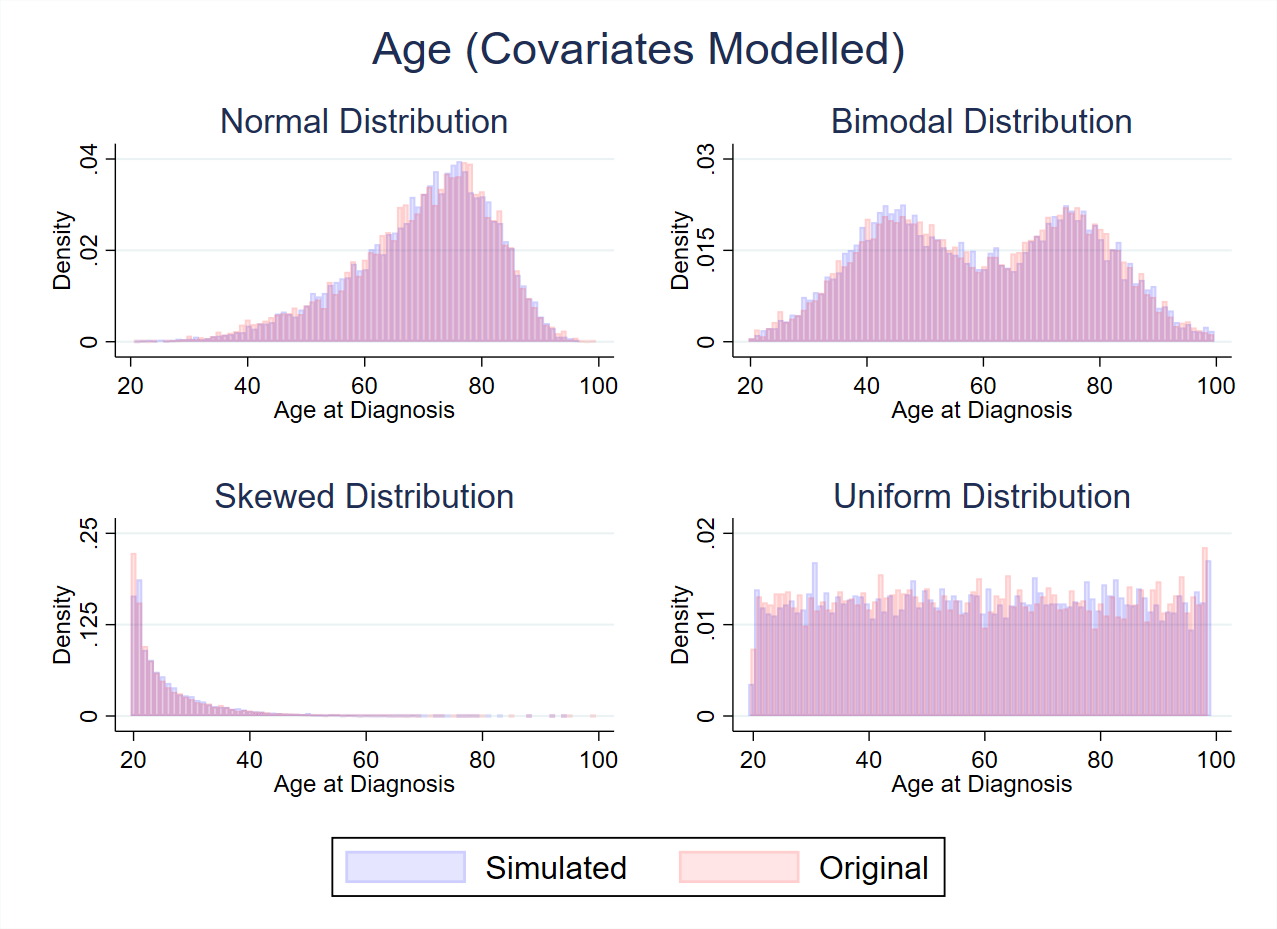


Figure 2: Demonstration of age distribution reconstruction methods for assorted distributions (Normal, Bimodal, Skewed and Uniform) where age is dependent on other variables

Figure 1 provides a visual demonstration of the effectiveness of the inverse-rank-based transformation method to reconstruct an age distribution accurately without incorporating any other covariate information into the regression model for age. Figure 2 shows the methods where other covariates (stage, sex, subsite, year of diagnosis) are included in the predictive regression model. As shown in table 1, both the “age only” and “age (covariates modelled)” do an effective job at recreating the marginal age distribution and key metrics found in the original data, across the assorted distributions being considered.

Table 1: Age distribution comparison details for "age only" and "age (covariate modelled)" for Normal, Bimodal, Skewed and Uniform distributions

|  | Obs | Mean | Std. dev. | Min | Max | Skewness | Kurtosis |
| --- | --- | --- | --- | --- | --- | --- | --- |
| AGE ONLY | | | | | | | |
| **Normal** | | | | | | | |
| Original | 9087 | 69.75 | 12.26 | 12 | 99 | -0.736 | 3.40 |
| Simulated | 9087 | 69.86 | 12.35 | 9.88 | 98.51 | -0.754 | 3.49 |
| **Bimodal** | | | | | | | |
| Original | 9087 | 59.90 | 17.79 | 10.39 | 107.93 | -0.001 | 2.04 |
| Simulated | 9087 | 59.61 | 17.91 | 5.74 | 111.04 | -0.024 | 2.03 |
| **Skewed** | | | | | | | |
| Original | 9087 | 26.12 | 8.47 | 20 | 106.17 | 2.66 | 12.91 |
| Simulated | 9087 | 26.18 | 8.64 | 19.47 | 105.57 | 2.76 | 13.72 |
| **Uniform** | | | | | | | |
| Original | 9087 | 59.55 | 22.74 | 20.00 | 98.99 | 0.005 | 1.81 |
| Simulated | 9087 | 59.30 | 22.72 | 20.02 | 99.34 | 0.024 | 1.81 |
| AGE (COVARIATES MODELLED) | | | | | | | |
| **Normal** | | | | | | | |
| Original | 9087 | 69.75 | 12.26 | 12 | 99 | -0.736 | 3.40 |
| Simulated | 9087 | 69.77 | 12.13 | 13.23 | 103.14 | -0.737 | 3.46 |
| **Bimodal** | | | | | | | |
| Original | 9087 | 59.91 | 17.86 | 7.23 | 106.07 | 0.018 | 2.05 |
| Simulated | 9087 | 59.87 | 17.81 | 9.25 | 108.18 | 0.025 | 2.04 |
| **Skewed** | | | | | | | |
| Original | 9087 | 26.08 | 8.57 | 20 | 99.77 | 2.684 | 12.85 |
| Simulated | 9087 | 25.94 | 8.32 | 19.34 | 104.74 | 2.704 | 13.55 |
| **Uniform** | | | | | | | |
| Original | 9087 | 59.62 | 22.69 | 20.01 | 98.99 | -0.009 | 1.84 |
| Simulated | 9087 | 59.38 | 22.61 | 19.47 | 99.30 | 0.002 | 1.85 |

**Stata Code to Create Simulated Age Distributions:**

1 ********************************************************************************

2 ***************************AGE NOT DEPENDENT*************************

3 ********************************************************************************

4 *UNIFORM DISTRIBUTION

5

6 use "https://www.pclambert.net/data/colon", clear

7 keep if year8594==1

8 gen female=sex-1

9

10 replace age=runiform(20,99)

11

12 egen rank = rank(age), unique

13 global N = _N

14 gen InvRankNormAge = invnormal((rank - 0.5)/${N})

15

16 rcsgen InvRankNormAge, df(5) gen(rcs) orthog

17 matrix Rmat=r(R)

18 local knots=r(knots)

19 regress age rcs*

20

21 gen U = rnormal()

22 drop rcs*

23 rcsgen U, knot(`knots') gen(rcs) rmatrix(Rmat)

24 predict agenew

25 gen agenew2 = (rnormal(agenew, `e(rmse)'))

26

27 twoway (hist agenew2, discrete width(1) color(blue%10)) ///

28 (hist age, discrete width(1) color(red%10)), ///

29 graphregion(color(white)) ///

30 title("Uniform Distribution") ///

31 xlabel(20(20)100) xtitle("Age at Diagnosis") ///

32 ylabel(0(0.01)0.02) ///

33 legend(order(1 "Simulated" 2 "Original")) name(uniform_ageonly, replace)

34

35 ********************************************************************************

36 ********************************************************************************

37 ********************************************************************************

38 *BIMODAL DISTRIBUTION

39

40 use "https://www.pclambert.net/data/colon", clear

41 keep if year8594==1

42 gen female=sex-1

43

44 gen p = runiform()<0.5

45 replace age=p*rnormal(45,10) + (1-p)*rnormal(75,10)

46

47 egen rank = rank(age), unique

48 global N = _N

49 gen InvRankNormAge = invnormal((rank - 0.5)/${N})

50

51 rcsgen InvRankNormAge, df(5) gen(rcs) orthog

52 matrix Rmat=r(R)

53 local knots=r(knots)

54 regress age rcs*

55

56 gen U = rnormal()

57 drop rcs*

58 rcsgen U, knot(`knots') gen(rcs) rmatrix(Rmat)

59 predict agenew

60 gen agenew2 = (rnormal(agenew, `e(rmse)'))

61

62 drop if agenew2 > 100

63 drop if agenew2 < 20

64

65 drop if age > 100

66 drop if age < 20

67

68 twoway (hist agenew2, discrete width(1) color(blue%10)) ///

69 (hist age, discrete width(1) color(red%10)), ///

70 graphregion(color(white)) ///

71 title("Bimodal Distribution") ///

72 xlabel(20(20)100) xtitle("Age at Diagnosis") ///

73 ylabel(0(0.015)0.03) ///

74 legend(order(1 "Simulated" 2 "Original")) name(bimod_ageonly, replace)

75

76 ********************************************************************************

77 ********************************************************************************

78 ********************************************************************************

79 *NORMAL DISTRIBUTION

80

81 use "https://www.pclambert.net/data/colon", clear

82 keep if year8594==1

83 gen female=sex-1

84

85 egen rank = rank(age), unique

86 global N = _N

87 gen InvRankNormAge = invnormal((rank - 0.5)/${N})

88

89 rcsgen InvRankNormAge, df(5) gen(rcs) orthog

90 matrix Rmat=r(R)

91 local knots=r(knots)

92 regress age rcs*

93

94 gen U = rnormal()

95 drop rcs*

96 rcsgen U, knot(`knots') gen(rcs) rmatrix(Rmat)

97 predict agenew

98 gen agenew2 = (rnormal(agenew, `e(rmse)'))

99

100 drop if agenew2 > 100

101 drop if agenew2 < 20

102

103 drop if age > 100

104 drop if age < 20

105

106 twoway (hist agenew2, discrete width(1) color(blue%10)) ///

107 (hist age, discrete width(1) color(red%10)), ///

108 graphregion(color(white)) ///

109 title("Normal Distribution") ///

110 xlabel(20(20)100) xtitle("Age at Diagnosis") ///

111 ylabel(0(0.02)0.04) ///

112 legend(order(1 "Simulated" 2 "Original")) name(normal_ageonly, replace)

113

114 ********************************************************************************

115 ********************************************************************************

116 ********************************************************************************

117 *SKEWED DISTRIBUTION

118

119 use "https://www.pclambert.net/data/colon", clear

120 keep if year8594==1

121 gen female=sex-1

122

123 replace age=runiform(20,99)

124

125 replace age = rgamma(0.5,0.012)*1000

126

127 egen rank = rank(age), unique

128 global N = _N

129 gen InvRankNormAge = invnormal((rank - 0.5)/${N})

130

131 rcsgen InvRankNormAge, df(5) gen(rcs) orthog

132 matrix Rmat=r(R)

133 local knots=r(knots)

134 regress age rcs*

135

136 gen U = rnormal()

137 drop rcs*

138 rcsgen U, knot(`knots') gen(rcs) rmatrix(Rmat)

139 predict agenew

140 gen agenew2 = (rnormal(agenew, `e(rmse)'))

141

142

143 gen age2 = age + 20

144 gen agenew3 = agenew2 + 20

145 drop if agenew3 < 20

146 drop if agenew3 > 100

147 drop if age2 > 100

148

149

150 twoway (hist agenew3, discrete width(1) color(blue%10)) ///

151 (hist age2, discrete width(1) color(red%10)), ///

152 graphregion(color(white)) ///

153 title("Skewed Distribution") ///

154 xtitle("Age at Diagnosis") xlabel(20(20)100) ///

155 ylabel(0(0.125)0.25) ///

156 legend(order(1 "Simulated" 2 "Original")) name(skewed_ageonly, replace)

157

158

159 grc1leg normal_ageonly bimod_ageonly skewed_ageonly uniform_ageonly, ///

160 graphregion(color(white)) title("Age Only") name(ageonly_comb, replace)

161

162

163 ********************************************************************************

164 ***********************AGE DEPENDENT ON COVARIATES*************

165 ********************************************************************************

166 *UNIFORM DISTRIBUTION

167

168 use "https://www.pclambert.net/data/colon", clear

169 keep if year8594==1

170 gen female=sex-1

171

172 replace age=runiform(20,99)

173

174 egen rank = rank(age), unique

175 global N = _N

176 gen InvRankNormAge = invnormal((rank - 0.5)/${N})

177

178 rcsgen InvRankNormAge, df(5) gen(rcs) orthog

179 matrix Rmat=r(R)

180 local knots=r(knots)

181 regress age rcs* i.stage##i.female ///

182 i.yydx##i.female i.female##i.subsite ///

183 i.subsite##i.stage i.subsite##i.yydx

184

185 gen U = rnormal()

186 drop rcs*

187 rcsgen U, knot(`knots') gen(rcs) rmatrix(Rmat)

188 predict agenew

189 gen agenew2 = (rnormal(agenew, `e(rmse)'))

190

191 twoway (hist agenew2, discrete width(1) color(blue%10)) ///

192 (hist age, discrete width(1) color(red%10)), ///

193 graphregion(color(white)) ///

194 title("Uniform Distribution") ///

195 xlabel(20(20)100) xtitle("Age at Diagnosis") ///

196 ylabel(0(0.01)0.02) ///

197 legend(order(1 "Simulated" 2 "Original")) name(uniform_agecond, replace)

198

199 ********************************************************************************

200 ********************************************************************************

201 ********************************************************************************

202 *BIMODAL DISTRIBUTION

203

204 use "https://www.pclambert.net/data/colon", clear

205 keep if year8594==1

206 gen female=sex-1

207

208 gen p = runiform()<0.5

209 replace age=p*rnormal(45,10) + (1-p)*rnormal(75,10)

210

211 egen rank = rank(age), unique

212 global N = _N

213 gen InvRankNormAge = invnormal((rank - 0.5)/${N})

214

215 rcsgen InvRankNormAge, df(5) gen(rcs) orthog

216 matrix Rmat=r(R)

217 local knots=r(knots)

218 regress age rcs* i.stage##i.female ///

219 i.yydx##i.female i.female##i.subsite ///

220 i.subsite##i.stage i.subsite##i.yydx

221

222 gen U = rnormal()

223 drop rcs*

224 rcsgen U, knot(`knots') gen(rcs) rmatrix(Rmat)

225 predict agenew

226 gen agenew2 = (rnormal(agenew, `e(rmse)'))

227

228 drop if agenew2 > 100

229 drop if agenew2 < 20

230

231 drop if age > 100

232 drop if age < 20

233

234 twoway (hist agenew2, discrete width(1) color(blue%10)) ///

235 (hist age, discrete width(1) color(red%10)), ///

236 graphregion(color(white)) ///

237 title("Bimodal Distribution") ///

238 xlabel(20(20)100) xtitle("Age at Diagnosis") ///

239 ylabel(0(0.015)0.03) ///

240 legend(order(1 "Simulated" 2 "Original")) name(bimod_agecond, replace)

241

242 ********************************************************************************

243 ********************************************************************************

244 ********************************************************************************

245 *NORMAL DISTRIBUTION

246

247 use "https://www.pclambert.net/data/colon", clear

248 keep if year8594==1

249 gen female=sex-1

250

251 egen rank = rank(age), unique

252 global N = _N

253 gen InvRankNormAge = invnormal((rank - 0.5)/${N})

254

255 rcsgen InvRankNormAge, df(5) gen(rcs) orthog

256 matrix Rmat=r(R)

257 local knots=r(knots)

258 regress age rcs* i.stage##i.female ///

259 i.yydx##i.female i.female##i.subsite ///

260 i.subsite##i.stage i.subsite##i.yydx

261

262 gen U = rnormal()

263 drop rcs*

264 rcsgen U, knot(`knots') gen(rcs) rmatrix(Rmat)

265 predict agenew

266 gen agenew2 = (rnormal(agenew, `e(rmse)'))

267

268 drop if agenew2 > 100

269 drop if agenew2 < 20

270

271 drop if age > 100

272 drop if age < 20

273

274 twoway (hist agenew2, discrete width(1) color(blue%10)) ///

275 (hist age, discrete width(1) color(red%10)), ///

276 graphregion(color(white)) ///

277 title("Normal Distribution") ///

278 xlabel(20(20)100) xtitle("Age at Diagnosis") ///

279 ylabel(0(0.02)0.04) ///

280 legend(order(1 "Simulated" 2 "Original")) name(normal_agecond, replace)

281

282 ********************************************************************************

283 ********************************************************************************

284 ********************************************************************************

285 *SKEWED DISTRIBUTION

286

287 use "https://www.pclambert.net/data/colon", clear

288 keep if year8594==1

289 gen female=sex-1

290

291 replace age=runiform(20,99)

292

293 replace age = rgamma(0.5,0.012)*1000

294

295 egen rank = rank(age), unique

296 global N = _N

297 gen InvRankNormAge = invnormal((rank - 0.5)/${N})

298

299 rcsgen InvRankNormAge, df(5) gen(rcs) orthog

300 matrix Rmat=r(R)

301 local knots=r(knots)

302 regress age rcs* i.stage##i.female ///

303 i.yydx##i.female i.female##i.subsite ///

304 i.subsite##i.stage i.subsite##i.yydx

305

306 gen U = rnormal()

307 drop rcs*

308 rcsgen U, knot(`knots') gen(rcs) rmatrix(Rmat)

309 predict agenew

310 gen agenew2 = (rnormal(agenew, `e(rmse)'))

311

312

313 gen age2 = age + 20

314 gen agenew3 = agenew2 + 20

315 drop if agenew3 < 20

316 drop if agenew3 > 100

317 drop if age2 > 100

318

319

320 twoway (hist agenew3, discrete width(1) color(blue%10)) ///

321 (hist age2, discrete width(1) color(red%10)), ///

322 graphregion(color(white)) ///

323 title("Skewed Distribution") ///

324 xtitle("Age at Diagnosis") xlabel(20(20)100) ///

325 ylabel(0(0.125)0.25) ///

326 legend(order(1 "Simulated" 2 "Original")) name(skewed_agecond, replace)

327

328

329 grc1leg normal_ageonly bimod_ageonly skewed_ageonly uniform_ageonly, ///

330 graphregion(color(white)) title("Age (Covariates Modelled)") ///

331 name(agemod_comb, replace)
